# Efficient TALEN-mediated gene targeting of chicken primordial germ cells

**DOI:** 10.1242/dev.145367

**Published:** 2017-03-01

**Authors:** Lorna Taylor, Daniel F. Carlson, Sunil Nandi, Adrian Sherman, Scott C. Fahrenkrug, Michael J. McGrew

**Affiliations:** 1 The Roslin Institute and Royal Dick School of Veterinary Studies, University of Edinburgh, Easter Bush Campus, Midlothian EH25 9RG, UK; 2 Recombinetics Inc, 1246 University Avenue West, Suite 300, Saint Paul, MN 55104, USA

**Keywords:** TALEN, Primordial germ cell, Avian, Transgenic knockout, Chicken, DDX4

## Abstract

In this work we use TALE nucleases (TALENs) to target a reporter construct to the *DDX4* (*vasa*) locus in chicken primordial germ cells (PGCs). Vasa is a key germ cell determinant in many animal species and is posited to control avian germ cell formation. We show that TALENs mediate homology-directed repair of the *DDX4* locus on the Z sex chromosome at high (8.1%) efficiencies. Large genetic deletions of 30 kb encompassing the entire *DDX4* locus were also created using a single TALEN pair. The targeted PGCs were germline competent and were used to produce *DDX4* null offspring. In *DDX4* knockout chickens, PGCs are initially formed but are lost during meiosis in the developing ovary, leading to adult female sterility. TALEN-mediated gene targeting in avian PGCs is therefore an efficient process.

## Abstract (precis)

**Summary:** TALE nucleases are used to target the *DDX4* (*vasa*) locus in chicken primordial germ cells and generate *DDX4* knockouts, which provide insights into DDX4 function in early chick development.

## INTRODUCTION

The chicken embryo is an established model for studying the genetic pathways regulating early patterning events and lineage commitment and also provides a comparative model for mammalian embryogenesis (Stern, 2005). In addition, the chicken is a key agricultural commodity, accounting for 30% of worldwide meat production and 1.2 billion eggs yearly [2012, Food and Agriculture Organization of the United Nations (http://www.fao.org/faostat)]. Therefore, the ability to precisely genetically edit the chicken genome will not only allow the investigation of key developmental signalling pathways in avian species but also the examination of genes involved in egg production, disease susceptibility and resistance with a view to promoting sustainability and biosecurity in both livestock and poultry production (Tizard et al., 2016; Whyte et al., 2016).

Genetic manipulation of avian species has lagged behind that of mammals owing to the complexity of the avian egg and the lack of germline-competent embryonic stem cell lines (Hunter et al., 2005). However, in sharp contrast to other vertebrate species, primordial germ cells (PGCs) from chicken can be propagated *in vitro* in suspension and maintain germline competence when transplanted back into donor embryos (van de Lavoir et al., 2006).

Vasa, a DEAD box RNA helicase originally identified in *Drosophila*, is essential for proper germ cell formation in multiple species (Schupbach and Wieschaus, 1986; Komiya et al., 1994; Gruidl et al., 1996; Kawasaki et al., 1998; Knaut et al., 2000). Moreover, in *Drosophila*, *C. elegans* and *D. rerio*, Vasa is essential for oogenesis, whereas in mice and basally branching insects Vasa homologues are necessary for male germ cells to progress through spermatogenesis (Kawasaki et al., 1998; Styhler et al., 1998; Tanaka et al., 2000; Kuramochi-Miyagawa et al., 2010; Ewen-Campen et al., 2013; Hartung et al., 2014). The divergent role of Vasa between different species is likely to be due to the differing mechanisms of PGC specification adopted by these species. In *Drosophila*, *C. elegans* and *D. rerio*, germ cell fate is acquired through the inheritance of maternal determinants, Vasa being one of these, and PGCs are present at the start of embryogenesis. By contrast, in mice, urodele amphibians and field crickets, PGCs arise from the mesoderm during mid-embryogenesis as a result of signalling cues (reviewed by Extavour and Akam, 2003). Expression of the chicken *vasa* homologue *DDX4* (also known as *CVH*) marks the chicken germ cell lineage at the earliest stages of embryonic development and therefore is hypothesised to be a maternal determinant for formation of the germ cell lineage (Tsunekawa et al., 2000). As a consequence, we would expect *DDX4* to play a role in oogenesis in the chicken.

Here, we use transcriptional activator-like effector (TALE) nucleases (TALENs) to knockout the *DDX4* locus in chickens, with the aim of demonstrating efficient targeting of genes important for development of the germ cell lineage. TALENs are synthetic transcription factors that can be modularly assembled into functional dimers that will target and cleave specific DNA sequences (usually 14-17 bp sequences for each module) in the target genome (Bogdanove and Voytas, 2011). Genomic cleavage can result in non-homologous end joining (NHEJ) that can cause small deletions or insertions (indels) at the target cleavage site. Introduction of a region of homology surrounding the cleavage site can result in homology-directed repair (HDR) that will lead to the incorporation of an exogenous DNA sequence into the target site of the genome. Classical gene targeting by homologous recombination has been demonstrated in cultured chicken PGCs (Schusser et al., 2013). Using CRISPR/Cas9, however, the frequency of homologous recombination in PGCs was greatly increased (Dimitrov et al., 2016). Both CRISPR and TALEN vectors have been used to generate indels in chicken PGCs and the resulting offspring (Park et al., 2014; Oishi et al., 2016), and to modify the genome of other vertebrate species (Tesson et al., 2011; Carlson et al., 2012, 2016; Tan et al., 2013; Zu et al., 2013).

Using TALEN-mediated homologous recombination we efficiently targeted the chicken *DDX4* locus. In contrast to the phenotype previously observed in mice, female chickens were sterile and contained no detectable follicles post-hatch. Examination of early embryos revealed that the germ cell lineage was initially formed but female PGCs were subsequently lost during meiosis. This study demonstrates the utility of TALENs in genome targeting of poultry and the conserved function of the *DDX4* gene in germ cell development and oogenesis.

## RESULTS

**Fig. 1.**
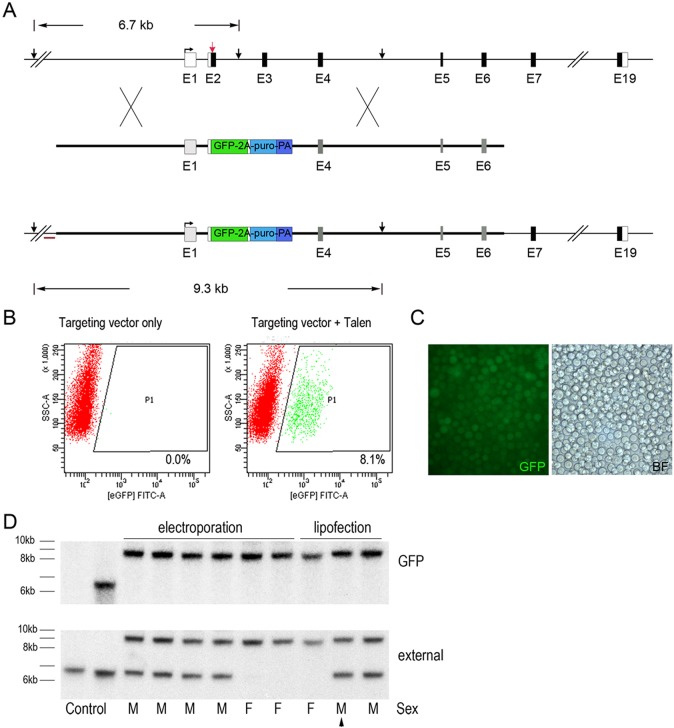
**Targeting of the chicken *DDX4* locus using TALENs.** (A) Overview of targeting strategy to generate a knockout/knock-in *DDX4* allele. The TALEN pair cleavage site is indicated (red arrow). Protein-coding exons are indicated by black boxes. Vertical black arrows, *Mfe*I sites. Red bar, external probe for Southern blot. (B) Targeting efficiencies of cultured PGCs. PGCs (male) were transfected with the targeting vector with or without the TALEN constructs, selected with puromycin for 2 days to enrich for transfected cells, cultured for 2 weeks and analysed by flow cytometry. The data represent one of two independent experiments with similar results. (C) Targeted GFP^+^ PGCs in culture after selection with puromycin. BF, bright-field. (D) Southern blot analysis of the GFP-puro targeted allele. For the external probe the expected fragment sizes are: wild-type, 6.7 kb; targeted; 11.7 kb. For the internal GFP probe the expected fragment size is: targeted, 11.7 kb. Genotype of cells is indicated by M (male) and F (female). Arrowhead, cell line used to generate targeted chicken (see text for details).

To target the *DDX4* locus we utilised TALEN-stimulated HDR to recombine a GFP-2a-puromycin transgene targeting vector into the *DDX4* locus, replacing exon 2 and 3 of the endogenous *DDX4* locus (Fig. 1A). A TALEN pair was designed that cleaved exon 2 of the *DDX4* locus immediately downstream of the ATG start codon. The targeting vector contained homology arms of 2.9 and 4.3 kb and fused the GFP-2a-puromycin reporter gene to the endogenous ATG codon. The correctly targeted locus expresses GFP-2a-puromycin under control of the endogenous *DDX4* regulatory regions, and a poly(A) termination signal terminates transcription prior to exon 4. The TALEN pair and the targeting vector were first transfected into PGCs and transiently selected (48 h) with puromycin to eliminate untransfected cells. PGCs were subsequently propagated in culture for 2 weeks and examined by flow cytometry to identify cells stably expressing the GFP transgene (Fig. 1B). PGCs transfected with the targeting vector alone did not express GFP. This is consistent with previous results that demonstrated that randomly integrated DNA vectors lacking insulator elements could not be stably selected in cultured PGCs (Leighton et al., 2008; Macdonald et al., 2012b). By contrast, 8.1% of the PGCs stably expressed GFP when co-transfected with the TALEN pair.

To demonstrate that the GFP transgene was correctly integrated into the *DDX4* locus, a Southern blot analysis was performed. Several independent transfections of male and female PGC lines were carried out and for each transfection the PGCs were selected with puromycin and genomic DNA was isolated (Fig. 1C). The *DDX4* gene is located on the Z chromosome and male (Z^GFP^Z) PGCs contain two chromosomal copies of the gene, whereas female (Z^GFP^W) PGCs are hemizygous and contain a single copy of the *DDX4* gene. Multiple targeting events are likely to occur during each transfection as clonal colonies were not selected. The intensity of the bands in the Southern blot indicates that a single allele was targeted in male cells and the female cells were hemizygous knockouts (Fig. 1D). Furthermore, RT-PCR analysis of a targeted female PGC line did not detect expression from the endogenous *DDX4* locus (Fig. S1, see supplementary Materials and Methods).

It has been reported that targeted insertion using site-specific nucleases can produce a targeted gene locus and disruption of the second chromosomal locus by NHEJ (Merkle et al., 2015). To address this concern, we sequenced the genomic locus of the non-targeted allele in the targeted male PGC lines and did not detect any instances of indels in the second chromosomal locus (Table S1). It is possible that we did not detect any NHEJ in the second allele because bi-allelic ablation of *DDX4* is lethal to male PGCs. This is unlikely as the targeted female PGC lines proliferated normally in culture (Fig. S1; data not shown). Accordingly, precise TALEN-mediated targeting of the *DDX4* locus was achieved.

**Fig. 2.**
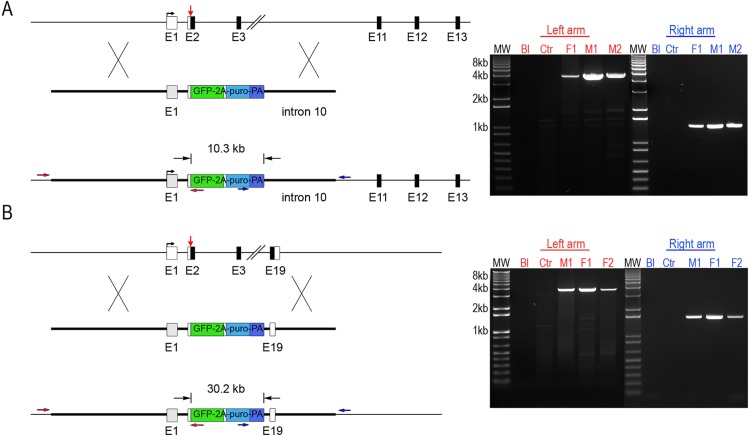
**Large genomic deletions of the *DDX4* locus using alternative homology arms.** (A,B, left) Strategy to generate larger deletions of the *DDX4* locus. The TALEN dimer is indicated by the red arrow and the positions of the right targeting arms are shown. The 5′ primer sets are shown in red and the 3′ primer sets in dark blue. (A, right) PCR genotype analysis of the *DDX4* targeted nine-exon deletion using external and internal amplification primers. Expected products: left arm, 3.6 kb; right arm, 1.8 kb. (B, right) PCR genotype analysis of the *DDX4* targeted 18-exon deletion using external and internal amplification primers. Expected products: left arm, 3.6 kb; right arm, 1.6 kb. Bl, blank; Ctr, control non-transgenic DNA; M1, M2, male cell line; F1, F2, female cell line.

We next asked if by varying the genomic location of the right homology arm larger deletions of the *DDX4* locus could be made while still co-transfecting with a single TALEN pair. The 4.3 kb right homology arm was replaced with 1.5 kb arms located after exon 10 or after exon 19, the final protein-encoding exon, of the *DDX4* gene. These targeting vectors would produce a genomic deletion of 10.3 kb or 30.2 kb, respectively, after correct integration into the *DDX4* locus (Fig. 2A,B, left). Following transfection, selection and expansion of PGCs, analysis of genomic DNA using primers located outside the homology arms revealed that the GFP-2a-puromycin transgene was precisely recombined into the endogenous *DDX4* locus and that a series of deletion alleles was produced encompassing the entire locus (Fig. 2B, right).

### Generation of targeted G_1_ offspring

**Fig. 3.**
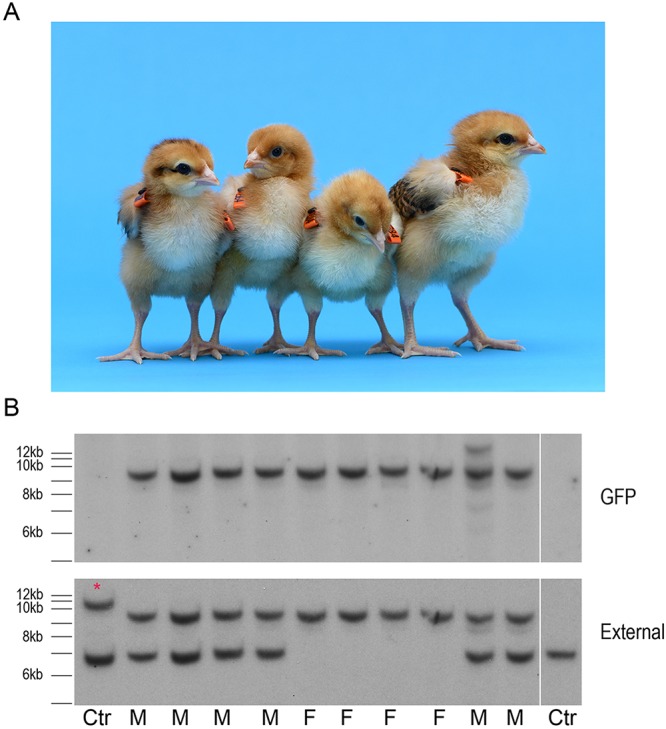
**Targeted knockout offspring produced from *DDX4* targeted PGCs.** (A) Targeted male and female G_1_ chicks. (B) Southern blot of control and individual G_1_ offspring. Fragment sizes are equivalent to those in Fig. 1. Asterisk marks a larger DNA fragment generated by restriction fragment length polymorphism in the control DNA sample.

Targeted male cells (Z^GFP^Z) (arrowhead, lane 10, Fig. 1D) were injected into surrogate host chicken embryos (day 2.5, stage 16 HH), incubated until hatching, and raised to sexual maturity. Two host male cockerels were assayed for the presence of the GFP reporter transgene in their semen and mated to wild-type hens (Table 1). One founder male did not transmit the targeted allele to offspring, whereas the second male generated 17 G_1_ targeted offspring (6%; Fig. 3A, Table 1). Southern blot analysis of genomic DNA from the G_1_ transgenic offspring demonstrated that the male chicks were heterozygous for the targeted allele and the female chicks were hemizygous mutant for *DDX4* (Fig. 3B). The Southern blot pattern exactly replicated the pattern seen in the PGC transfections (Fig. 1D), confirming that the PGCs were targeted at a single allele.

**Table 1.**
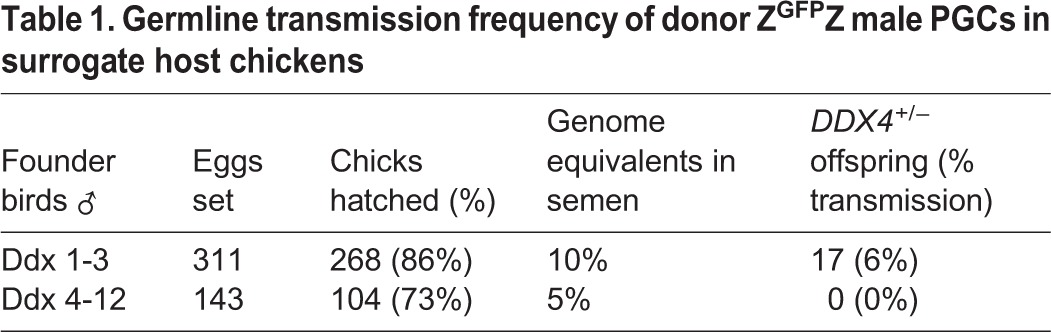
**Germline transmission frequency of donor Z^GFP^Z male PGCs in surrogate host chickens**

### *DDX4* is required for female fertility in birds

**Fig. 4.**
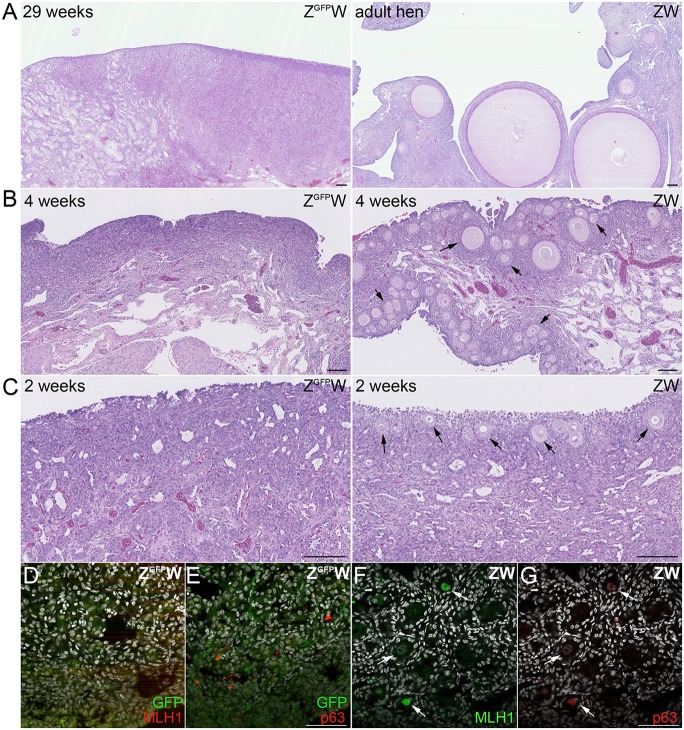
**No follicles are present in the cortex of post-hatch Z^GFP^W hens.** (A-C) H&E staining of representative post-hatch Z^GFP^W and ZW hens. Arrows indicate developing follicles. (C) Z^GFP^W, 0 follicles/field; ZW, 26 follicles/field; average of ten fields from four ovaries/genotype. Scale bars: 100 µm. (D-G) Immunostaining of 2-week post-hatch follicles for GFP and the germ cell meiotic markers p63 and MLH1. Nuclear stain, white. Arrows indicate developing follicles. Scale bars: 50 µm.

In commercial egg-laying hens, ovulation begins at week 18 post-hatch (PH) from a single ovary originating from the left gonad. The mature ovary contains several thousand small white follicles and 30-100 yellow or small yolky follicles (Gilbert et al., 1983). As *Ddx4* knockout female mice exhibit normal reproductive capacity (Tanaka et al., 2000), the G_1_ chickens were raised to sexual maturity with the intention to cross the females and males to generate homozygous Z^GFP^Z^GFP^ males. Unexpectedly, the seven hemizygous Z^GFP^W female G_1_ chickens did not enter into lay by 29 weeks PH. A morphological examination of the ovaries from these hens revealed that no white or yellow follicles were visible and no primary or secondary follicles were detected in sections (Fig. 4A, Fig. S2). To examine the earlier stages of ovarian development, a Z^GFP^Z heterozygote male was mated to wild-type ZW hens and ovaries of age-matched Z^GFP^W and ZW hatchlings were examined post-hatch. In the hatchlings, primary and secondary follicles surrounded by layered granulosa cells were present at 2 and 4 weeks PH (*n*=6, ZW), whereas no follicles were detected in the ovaries of the Z^GFP^W chicks (*n*=6; Fig. 4B,C). Similarly, immunostaining for GFP and germ cell markers of mature oocytes (p63 and MLH1) did not reveal any germ cells in the ovary at week 2 PH (Fig. 4D-G).

To identify whether the defect in oogenesis was due to ablation of the germ cell lineage, we examined early embryonic stages of the Z^GFP^W embryos. DDX4 protein marks the germ cell lineage at cleavage stages of embryonic development and *DDX4* RNA is expressed during PGC migration to the gonad and at all subsequent developmental stages (Tsunekawa et al., 2000). We found that GFP^+^ cells were present in Z^GFP^W gonads at day 6 and day 9 of incubation (Fig. S3A,B). These cells expressed the germ cell marker SSEA1. Immunostaining confirmed the lack of DDX4 protein in day 9 female PGCs of Z^GFP^W embryos (Fig. S3B). We examined the number of proliferative cells in the gonad of day 10.5 embryos to determine whether proliferation was compromised in the developing gonad in the absence of DDX4. There was a significant reduction in EdU^+^ PGCs in the developing cortex of Z^GFP^W gonads (Fig. S3C). These results show that germ cells initially form in Z^GFP^W chicken embryos and are lost at later developmental stages.

To determine if the Z^GFP^W germ cells entered meiosis, several meiotic markers were examined in the developing ovary. PGCs in the female gonad enter meiosis by day 16 of embryonic development and most oocytes reach the diplotene stage of meiosis I by day 7 PH (Smith et al., 2008; del Priore and Pigozzi, 2012). In control embryonic day 17 ZW gonads, germ cells correctly express the meiotic markers SCP3, γH2AX and MLH1 (Fig. S4). By contrast, SCP3 was not detected at this stage, and germ cell number was reduced in the Z^GFP^W ovary. By embryonic day 19, Z^GFP^W germ cells expressed SCP3 at levels comparable to wild-type ZW germ cells, although visibly fewer germ cells were present in the cortex of Z^GFP^W gonads (Fig. S4). The widespread expression of MLH1 indicated that many germ cells in both Z^GFP^W and ZW gonads had reached the pachytene stage of meiosis I. Expression of SCP1 was also detected in embryonic day 19 Z^GFP^W embryos (Fig. S4). To determine if the remaining Z^GFP^W germ cells progressed to later stages of meiosis, chicks were examined at 3 days PH. In chickens, the majority of germ cells have entered the pachytene stage of meiosis at day 3 PH (Hughes, 1963; del Priore and Pigozzi, 2012). In control ZW day 3 hatchlings, SCP3^+^ MLH1^+^ germ cells were present in the cortex of the ovary and SCP3 was expressed in a punctate manner indicative of the pachytene stage of meiosis (Fig. S4) (Guioli et al., 2012). By contrast, the majority of the GFP^+^ cells were located in the medulla of the Z^GFP^W day 3 PH ovary. The limited number of GFP^+^ cells found in the cortex were MLH1^+^, but very few expressed SCP3. These data are in agreement with a defect in progressing beyond the pachytene stage of meiosis, leading to a post-hatch loss of germ cells in the Z^GFP^W ovary.

## DISCUSSION

These results demonstrate that TALEN-stimulated HDR in germ cells is highly efficient and opens future avenues for investigation of gene function in birds and for the introduction of production traits. Large genomic deletions have been achieved using two site-specific nucleases or CRISPR vectors, but to our knowledge this is the first large genetic deletion produced using a single nuclease pair. Genetic deletions through altered placement of homology arms will be useful to create a series of genetic deletions at loci of interest. CRISPR-stimulated HDR in chicken germ cells has been reported but the recombination efficiency was much lower than reported here (Dimitrov et al., 2016). As *DDX4* is expressed in PGCs, it is possible that this genomic locus may be highly accessible to site-specific nucleases and amenable to gene targeting. Additionally, the cell culture medium used in this report supports rapid cell proliferation (Whyte et al., 2015), which might lead to a much higher frequency of HDR. We did not observe a phenotype in cultured female targeted PGCs or at early embryonic stages in Z^GFP^W embryos. Early PGCs express the DEAD box genes *DDX43* and *DDX25* (Jean et al., 2015), and it is possible that these proteins replace *DDX4* at early developmental stages.

The precise role of DDX4 in meiosis remains unknown. The mouse *Ddx4* knockout led to male sterility; male mouse PGCs enter meiosis but do not express diplotene markers and undergo apoptosis. Proliferation of *Ddx4*^−/−^ PGCs was also severely compromised in the early mouse gonad, similar to what was observed here in the forming ovary of Z^GFP^W embryos (Tanaka et al., 2000). Ddx4 is thought to function in amplifying the translation of a number of proteins needed for meiotic progression and the assembly of cytoplasmic granules in germ cells, which are potential RNA-processing centres (Aravin et al., 2009; Kuramochi-Miyagawa et al., 2010). Future experiments will address the function of DDX4 in chicken germ cell meiosis.

## MATERIALS AND METHODS

### PGC culture

PGC line derivation and culture were carried out as described (Whyte et al., 2015). Briefly, 1 μl blood isolated from a stage 16 HH embryo (Hamburger and Hamilton, 1992) was placed in culture medium containing 1× B-27 supplement (Thermo Fisher Scientific), 0.15 mM CaCl_2_, 2.0 mM GlutaMax (Thermo Fisher Scientific), 1× non-essential amino acids (Thermo Fisher Scientific), 0.1 mM β-mercaptoethanol, 1× EmbryoMax nucleosides (Merck Millipore), 1.2 mM pyruvate (Thermo Fisher Scientific), 0.2% ovalbumin (Sigma) and 0.01% sodium heparin (Sigma). 25 ng/ml activin A (Peprotech), 4 ng/ml FGF2 (R&D Systems) and 5 µg/ml ovotransferrin (Sigma) were added to Avian Knockout DMEM (osmolality: 250 mOsmol/kg, 12.0 mM glucose, calcium chloride free; Thermo Fisher Scientific, a custom modification of Knockout DMEM). Chicken serum at 0.2% (Biosera) was added to this medium to produce FAOTcs medium. A male and a female PGC line were derived in FAOTcs medium and expanded to 2.5×10^5^ cells in 5 weeks before use in targeting experiments.

### TALEN design and construction

All TALENs were designed using TALE-NT software and assembled using methods described in Cermak et al. (2011). Design and construction of ggVASAe1.1 is described in Carlson et al. (2012) using the pC-GoldyTALEN (Addgene ID 38143). The TALEN pair creating a cleavage site 15 bp 5′ to the ATG of *DDX4* was designed (DDX1.1 targeting vector) with the following binding sites: left monomer (sense), GCTAACGTGCTCCTGGTCCT; right monomer (sense), ATTCGCTATGGAGGAGG. A 2.9 kb genomic fragment upstream of exon 2 of the *DDX4* gene was PCR amplified from ISA Brown genomic DNA and cloned upstream of a GFP-2a-puromycin-poly(A) expression cassette (Hockemeyer et al., 2011) by converting the endogenous ATG to an *Nco*I site (outer primers, 5′-CTGGTAGAGAGCATTACAAAAGTC-3′ and 5′-GTGTCCCAGTCCTCCTCCATAG-3′; inner *Nco*I primer, 5′-AACCATGGCGAATGCCAGCAGCCCA-3′). A 4.3 kb downstream PCR fragment containing exons 4-6 was cloned into a *Bam*HI site downstream of the poly(A) site to create pddx4-GFP-polyA-exon4 using nested primers (outer primers, 5′-CTCCTTGGCCCCATTAACAGA-3′ and 5′-GTTTTGTGCCATGACCACTG-3′; inner primers, 5′-GGGGCCCAGAAGTTCTCCTTA-3′ and 5′-TTGGGCCCAAATCCACGGTGCAATATCC-3′). This right arm was replaced by digesting with *Bam*HI and the following right arms were used: a 1.5 kb PCR fragment containing intron 10 termed pddx4-GFP-polyA-intron10 (primers 5′-TAGTTGGATGCCTCAGACTTCA-3′ and 5′-ATTGCAAGTGGAGCTTCAAGA-3′) and a 1.5 kb PCR fragment downstream of exon 19 termed pddx4-GFP-polyA-3pUTR (primers 5′-GAAGGCAAAAGCCATTTTCA-3′ and 5′-CCCTTCTAAACCCTGCAATTC-3′) using Phusion HF polymerase (New England Biolabs).

### PGC transfection and electroporation

1 µg TALEN vector pair 1.1 (0.5 µg each of left and right) and 1 µg targeting vector were co-transfected into PGCs using DIMRIE transfection reagent (Thermo Fisher Scientific) as previously described (Macdonald et al., 2012a). Briefly, 1×10^5^ PGCs were washed in Optimem I (Thermo Fisher Scientific), mixed with the DNA and transfection reagent and transfected in suspension for 6 h. PGCs were then centrifuged and resuspended in FACS medium. For electroporation, 1×10^5^ PGCs were centrifuged and resuspended in 10 µl solution R containing 1 μg DNA (0.5 µg TALEN vector pair 1.1 and 0.5 µg targeting vector) and electroporated using a Neon electroporator (Thermo Fisher Scientific) at 850 V, 50 ms pulse. To measure targeting efficiencies, PGCs were selected 24 h post-transfection with 0.6 µg/ml puromycin for 48 h, then washed to remove all puromycin and further cultured for 2 weeks to eliminate transient GFP fluorescence. To select stably transfected cells, PGCs were selected at 4 days post-transfection using 0.3 µg/ml puromycin treatment over a 2 week period. PGCs from each individual transfection were then expanded in culture for 29 days to 8×10^5^ cells and cryopreserved using Avian Knockout DMEM/B-27 supplement containing 5% DMSO and 4% chicken serum and frozen for 9 months before use in germline transmission experiments. Genomic DNA was isolated from each individual transfection and used for Southern blot analysis.

### Immunohistochemistry

Tissues were fixed in formalin for paraffin sections followed by Haematoxylin and Eosin (H&E) staining or cryo-embedded and processed for immunofluorescence (Whyte et al., 2015). The number of follicles per field for 2-week PH ovaries was determined by counting one microscope field per slide for four slides from four different ovaries for each genotype. Samples were incubated with primary antibody in 5% goat serum overnight at 4°C. Details of antibodies are provided in the supplementary Materials and Methods.

### Flow cytometry

For flow cytometry analysis, PGCs were transiently selected with puromycin at 24 h post-transfection to enrich for transfected cells. PGCs were treated for 48 h then washed to remove puromycin. After culture for an additional 3 weeks, PGCs were analysed for GFP fluorescence using a FACSAria II (BD Biosciences) to identify stably integrated cells.

### Germline transmission

Y25 male-targeted cells (Novagen Brown) were thawed from storage at −150°C for 9 months and cultured for 4-8 days before injection into stage 16 HH surrogate host embryos in windowed eggs (Whyte et al., 2015). 3000-5000 PGCs were injected into the dorsal aorta, the shells were resealed with Parafilm and the eggs incubated at 37.7°C until hatching (McGrew et al., 2004; Nakamura et al., 2008). Four injection experiments were carried out. PCR screening for the GFP transgene in the semen of two founder male cockerels was performed and these were then bred to wild-type hens. Offspring were screened by PCR for the presence of the GFP transgene (McGrew et al., 2004). Genomic DNA was isolated from the blood of GFP^+^ G_1_ offspring and used for Southern blot analysis. Animal experiments were conducted under UK Home Office license.

### Southern blot analysis

Genomic DNA was isolated from blood samples or stably transfected PGCs (∼1×10^6^ cells) using a Flexigene Kit (Qiagen) and 5 µg was digested overnight with *Mfe*I. The DNA digests were resolved by gel electrophoresis and transferred via capillary action to Hybond N membrane (GE Healthcare). A GFP fragment (0.8 kb) or a 0.6 kb *DDX4* genomic DNA fragment (amplified using primers 5′-GACAAGCCATCACATACAAAGC-3′ and 5′-AAGGAAGCTGGGAGCTCTTC-3′) was labelled with [α-^32^P]dCTP using the DIG High Prime DNA Labelling and Detection Kit II (Roche) and used to hybridise the Southern blot.

To assay integration into the *DDX4* locus by PCR, the following nested primers were used: for the common left arm, outer primers (5′-CAGCACTGTTAAAGGGCACA-3′ and 5′-AAGTCGTGCTGCTTCATGTG-3′) and inner primers (5′-GCGCGCTTTGACATATTTTT-3′ and 5′-GGTCACGAGGGTGGGCCAG-3′); 11 kb right arm (5′-GCCTGAAGAACGAGATCAGC-3′ and 5′-TCCACTGCCATATGAGGACA-3′); 20 kb right arm (5′-GCCTGAAGAACGAGATCAGC-3′ and 5′-GGGGTTGGACTTAATCTCTGG-3′).
